# Improved nutrition in early life and pulse wave velocity and augmentation index in mid-adulthood: Follow-up of the INCAP Nutrition Supplementation Trial Longitudinal Study

**DOI:** 10.1371/journal.pone.0239921

**Published:** 2020-10-27

**Authors:** Maria F. Kroker-Lobos, Nicole D. Ford, Ines Gonzalez-Casanova, Reynaldo Martorell, Manuel Ramirez-Zea, Aryeh D. Stein

**Affiliations:** 1 INCAP Research Center for the Prevention of Chronic Diseases, Institute of Nutrition of Central America and Panama, Guatemala City, Guatemala; 2 Hubert Department of Global Health, Rollins School of Public Health Emory University, Atlanta, Georgia, United States of America; University of Tasmania, AUSTRALIA

## Abstract

Nutrition in pregnancy and early childhood affects later blood pressure and precursors of atherosclerosis, but its influence on arterial stiffness is unexplored. This study determines whether exposure to improved nutrition during early life influences Augmentation index (AI) and pulse wave velocity (PWV) in mid-adulthood. We included 1221 adults (37-54y) who participated in a cluster-randomized nutritional supplementation trial of a protein-energy beverage (*Atole*), conducted between 1969–1977 in Guatemala. The comparison group received *Fresco*, a low-calorie protein-free beverage. In 2015–17, we measured anthropometry (weight, height, and waist-to-height ratio); AI and PWV (using carotid—femoral tonometry); blood pressure; fasting plasma glucose and serum lipids; and sociodemographic characteristics. Based on patterns of exposure, we characterized participants as fully, partially or unexposed to the intervention from conception to their second birthday (the ‘first 1000 days’). We fit pooled and sex-specific models using intention-to-treat, difference-in-difference regression analysis to test whether exposure to the supplement in the first 1000 days was associated with AI and PWV in adulthood adjusting for basal and current sociodemographic variables and current life-style and cardio-metabolic risk factors. Prevalence of obesity in men and women was 39.6% and 19.6%, and prevalence of hypertension was 44.0% and 36.0%, respectively. Women had higher AI (34.4±9.6%) compared to men (23.0± 9.8%), but had similar PWV (7.60±1.13 m/s and 7.60±1.31, respectively). AI did not differ significantly across intervention groups. PWV was lower in individuals with full exposure to the supplement during the first 1000 days (-0.39m/s, 95% CI -0.87, 0.09; p = 0.1) compared to unexposed individuals. This difference was similar after adjusting for cardio-metabolic risk factors (-0.45m/s; 95%C-0.93, 0.01; p = 0.06). Exposure to improved nutrition during the first 1000 days was marginally associated with lower PWV, but not with AI.

## Introduction

Carotid-femoral pulse wave velocity (PWV) and augmentation index (AI) are measures of arterial stiffness and central arterial pressure, and thus are proxy indicators of arterial aging [1]. Increased stiffness, the loss of elasticity, in the arterial tree is a strong independent predictor of atherosclerosis [2, 3]. PWV and AI are independent predictors of mortality in the general population [4, 5] and they have been linked to age, hypertension, central obesity, heart rate, height, diabetes, and several lifestyle risk factors [6–9]. Degeneration of compliant elastin fibers and deposition of stiffer collagen in the arterial wall, which may start in childhood [10], are among the primary causes of age-related arterial stiffening [1].

Breastfeeding is associated with favorable brachial endothelial function, properties of the carotid arterial wall, and brachial blood pressure [11–13]. One study showed adverse effects of protein-enriched infant formula given during the first 9 months to children small for gestational age of life on blood pressure (6–8 years), when compared with standard formula [14]. Similarly, supplementation of preterm infants with cow’s milk during the first 5 years of life increased brachial blood pressure in adulthood [15]. Longitudinal studies in several low and middle income countries found evidence that growth failure is associated with elevated blood pressure in adulthood [16]. However, the role of nutrition in early life on later arterial stiffness remains largely unexplored, and there is no evidence on the effects of nutritional supplementation on arterial stiffness in undernourished populations.

Our objective was to explore whether improvements in nutrition in early life are associated with arterial stiffness in early middle age in a population growing up in a context of chronic undernutrition, and to assess whether this association is independent of attained height, mean blood pressure, and current lifestyle risk factors.

## Materials and methods

### Study population

**Fig 1:**
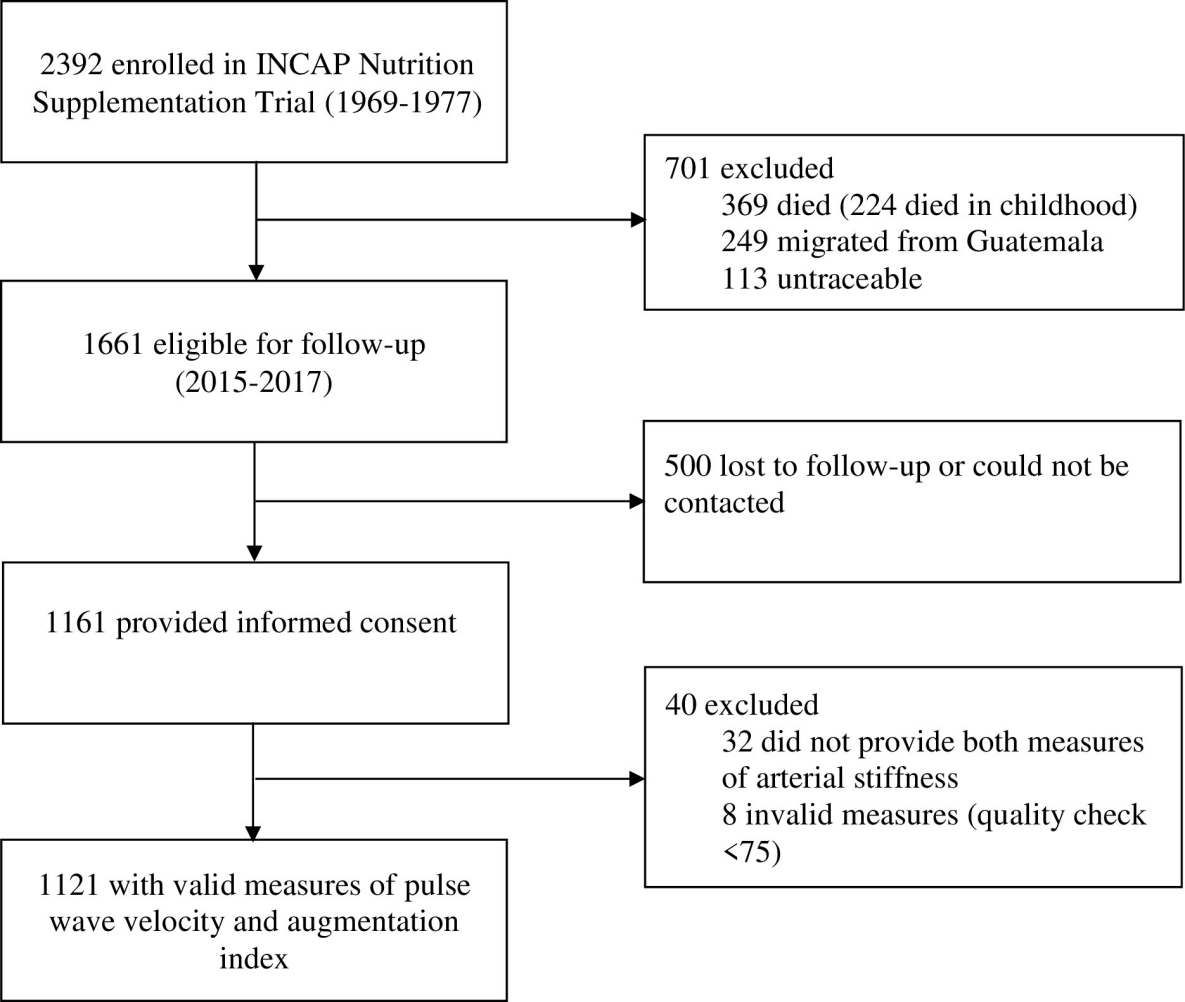
Enrollment, participation and final sample of the Institute of Nutrition and Central America and Panama (INCAP) Supplementation trial follow-up in 2015–2017.

The INCAP Nutrition Supplementation Trial Longitudinal Study is a follow-up of individuals who participated as children in the INCAP Nutrition Supplementation Trial, which was conducted in four communities in eastern Guatemala to study the effect of early-life improved nutrition on development and growth [17]. In the original trial, conducted between 1969 and 1977, two pairs of matched villages were randomized to *Atole*, a protein-energy beverage, or *fresco*, a protein-free, low-calorie beverage. The supplements were distributed in a centrally located feeding hall for 2–3 h during midmorning and midafternoon, including weekends. The beverage was prepared daily and supplement intake was recorded for pregnant and lactating women and children [17]. Details of the methods of the original supplementation trial and the nutritional composition of *Atole* and *Fresco* are published elsewhere [17]. Participating individuals have been followed prospectively; the present analysis uses data collected in the 2015–17 follow-up, which was designed to test the hypothesis that improved nutrition during the ‘first 1000 days’ (the period from conception to the second birthday) can attenuate the development of cardiometabolic disease. Fig 1 shows the trial profile. Out of 2392 participants enrolled in the original trial, 269 died, 249 migrated out of the country and 114 were untraceable. Among the 1661 participants living in Guatemala, 500 could not be contacted or declined to participate; 1161 individuals provided informed consent. Finally, 40 participants were excluded as they did not have both measures of arterial stiffness (either AI or PWV) or measures did not reach precision quality, resulting in a final sample size of 1121.

#### Measurements in the original study (1969–77)

In the original study, staff performed data collection at a research facility or in the participant´s home. The informant was the mother or other primary caretaker. Data used in the present paper include the child’s birth year and sex, and maternal height, schooling (grades completed), age at the childbirth, and household socio economic status (SES) at the time of the childbirth.

#### Measurements in the 2015–2017 follow-up

In the 2015–17 follow-up, trained staff collected data in a research facility in each village, in the nearby town of Sanarate, or in Guatemala City. Through face-to-face interviews, we obtained data on smoking and alcohol consumption (Yes/no, current status); socio-demographic characteristics, current residence (Guatemala City vs other), self-reporting of health conditions, and reported use of hypertension and diabetes medication.

*Anthropometry*. Standard procedures were applied to obtain duplicate measures of height, body weight, and waist circumference (WC) [18]. WC and height were measured to the nearest 0.1cm and weight to the nearest 100g. If the difference between the 1^st^ and 2^nd^ measures exceeded 500g for weight, 0.5 cm for height or 1 cm for WC, a third measure was taken. We calculated the average using the two closest measures. Body mass index (BMI) was calculated as weight in Kg divided by the squared of height in meters; BMI ≥ 25 kg/m^2^ was considered overweight and BMI> 30 kg/m^2^ was considered obese [19]. The waist-to-height ratio (WHtR), a marker of central obesity, was calculated as the ratio of WC and height, both in meters [20].

*Body composition*. We estimated total body water (TBW) using the deuterium oxide (D_2_O) dilution technique. Participants provided a saliva sample for determination of the background deuterium enrichment and then received an oral dose of 30 g of deuterium water. After 3 h, a second saliva sample was collected. Deuterium enrichment was analyzed through the Fourier Transform Infrared (FTIR, Shimadzu 8400S) spectroscopy in the range 2300–2800 cm^-1^. TBW was determined from the deuterium oxide dilution space, corrected for 4% exchange of deuterium with the non- aqueous compartment of the body. We calculated fat free mass (FFM) from TBW, assuming that FFM has a hydration constant of 0.732. Fat mass was estimated as the difference between total body weight and FFM [21].

*Peripheral blood pressure and mean blood pressure*. Using a cuff size appropriate for the participant’s arm size, seated systolic blood pressure (SBP) and diastolic blood pressure (DPB) were measured 3 times. The first reading was obtained after 5 minutes of seated rest and the 2^nd^ and 3^rd^ readings at 3 min intervals using a digital blood pressure monitor (Omron, Schaumburg IL, USA). If the difference between the second and third systolic or diastolic measurements were greater than >10 mmHg, a fourth measure was taken. The average of the two closest measurements was used without including the first measurement. We defined hypertension as systolic blood pressure ≥130 mmHg and/or diastolic blood pressure ≥80 mmHg and/or hypertension medication use [22]. Mean arterial blood pressure was calculated as = DBP + (SBP–DBP) / 3 [23].

*Blood lipids and type 2 diabetes*. Trained phlebotomists obtained venous blood samples in fasting conditions (at least 8 hours) and 120 minutes after a prandial challenge. Fasting triglycerides, high-density lipoprotein cholesterol (HDL-c), low-density lipoprotein cholesterol (LDL-c), glycated hemoglobin (HbA1c), and fasting and 2-h post-challenge glucose were measured by enzymatic colorimetric methods (Cobas C111 analyzer, ROCHE, Indiana, USA) at INCAP laboratories. We defined type 2 diabetes as fasting plasma glucose ≥126 mg/dL and/or post-challenge glucose ≥200 mg/dL and/or diabetes medication use [24].

*Arterial stiffness and central hemodynamic data*. We derived central systolic, central diastolic pressure, PWV, AI, and AI standardized at a constant heart rate (75bpm) from pulse wave analysis using a Sphygmocor Xcel (AtCot Medical, Itasca IL), recording the pressure waveforms at the carotid and femoral arteries. With the participant in a supine position, a trained and standardized physician placed a cuff around the femoral artery to capture the femoral waveform, and a tonometer was positioned at the base of the neck on the common carotid artery. The distance between the carotid and femoral arteries was measured over the body surface, and the velocity was determined by dividing pulse transit time by the distance. The software quantifies the quality of the captured peripheral (brachial) waveform in 0–100 scale. An overall quality value above or equal to 75 was used to define valid measures of PWV and AI as indicated by the device software.

We obtained ethical approval from the Institutional Review Boards of Emory University (Atlanta, GA) and INCAP (Guatemala City, Guatemala). All participants gave written informed consent before participation.

### Statistical methods

#### Nutrition exposure in early childhood

Our primary interest was exposure during first 1000 days, since this period is critical for linear growth, development, human capital and health outcomes in adulthood [25]. Children were exposed to supplementation (*Atole* or *Fresco*) at different ages, depending on their date of birth. Supplementation started on Jan 1^st^, 1969 in two villages and on May 1^st^, 1969 in the other two and ended on February 28, 1977 in all villages. We defined exposure to *Atole* supplementation as being full if children resided in the *Atole* villages and were fully exposed to the supplement from conception to age 2y (first 1000-days). We defined exposure as partial if children resided in the *Atole* villages and received the supplement partially during the first 1000-days. Finally, we defined children as unexposed if they resided in the *Fresco* villages or if they did not receive *Atole* during the first 1000-days. For these definitions a gestational period of 266 day was assumed.

#### Statistical analysis

General characteristics of the study population and distribution of outcomes, exposure and covariates were assessed by means and proportions for continuous and categorical data, respectively. Since there is limited evidence of arterial stiffness in Latin America and this is the first time that proxies of arterial stiffness were measured in a Central American population, medians and percentiles (10^th^, 50^th^, and 90^th^) of AI and PWV by sex and age were compared to references values [26, 27]. Sex-specific and pooled bivariate associations between measures of arterial stiffness and cardio-metabolic risk factors, age, residency and SES were calculated using linear regression.

We developed a socioeconomic index (SES) using household characteristics information and consumer durable goods captured in the sociodemographic questionnaires. We categorized SES into tertiles. Methods to derive the SES scores for this cohort are reported elsewhere [28].

#### Main analysis

To assess associations between exposure to *Atole* in early life and measures of systemic arterial stiffness in adulthood, we conducted difference-in-difference (DD) intention-to-treat analysis using generalized linear models. For the DD analysis, our primary interest was the interaction term between the exposure period (fully exposed during the first 1000 days, partially exposed in the first 1000 days, unexposed) and supplementation type (*Atole* vs *fresco*). This interaction term represents the differential effect of exposure to *Atole* compared with *fresco* during all or part of the first 1000 days, after subtraction of the difference between individuals exposed to *Atole* versus *Fresco* at other ages. The interaction term, therefore, is an estimate of the effect of exposure to *Atole* in the first 1000 days. Given the differences in morphology between men and women, we developed separate models for men and women. All pooled analyses were adjusted for sex. Standard errors were calculated with allowance for clustering within families.

For each outcome, we assessed four different models. The base model (model 1) included dummy variables for birth village to account for village fixed effects and supplementation type, age of exposure to the intervention (full or partial vs none), birth year (to control for any cohort effects), and the previously-described interaction between supplementation type and age at intervention. In model 2, covariates were selected based on previous findings in this cohort [29–31]. We added early-life characteristics, specifically SES in childhood and maternal factors including age at childbirth, height, and completed grades of schooling. In model 3, we added a set of current well-known confounders, such as sociodemographic characteristics including age; SES; residence; smoking and alcohol consumption; height; mean blood pressure; and hypertension medication use [2, 3, 7]. Finally, in model 4 we adjusted for several conditions to assess the potential mediation influence, such as BMI, central obesity, fat free mass, body fat percentage, blood lipids, and diabetes [6, 9].

In all models, heart rate was included as a co-variate except for models in which AI was standardized to 75 bmp [32, 33]. We tested for heterogeneity by sex through a third-order interaction term of sex, A*tole/Fresco* and exposure to supplementation.

Negative values of AI may occur in young men, and restricting analysis to individuals with positive values of AI has been proposed to avoid distortion of results [33]. In our sample, only four men had negative values of AI and were included in the analysis, since the estimates were not affected by their exclusion. Since AI is highly dependent on heart rate, we also conducted analyses using as outcome AI standardized to 75bpm. All p values were two-sided and statistical significance was set at p<0.05.

#### Missing data

There were 233 participants with missing information for maternal height (20.6%), 100 for post-prandial glucose (8.8%), 97 for SES at birth (8.6%); 40 for mother’s schooling (3.5%), 30 for fat mass and 28 for fat free mass (2.6% and 2.5%, respectively). Fewer than 1% of participants had missing values for blood lipids, fasting glucose, alcohol and smoking status, hypertension medication use, and blood pressure. To handle missing data, we assumed that incomplete data was missing at random, in order to perform multiple imputation (MI) with 50 imputations for each incomplete variable [34]. We imputed incomplete values using correlated variables with no missing such as sex, age, birth year, BMI, WHtR, height, waist circumference, heart rate, current residence, current SES, grades of schooling, PWV and AI. Then, we fit the desired linear regression models. We also obtained the fraction of missing information–FMI-, which measures the level of uncertainty about the values one would impute for current non-responders and we used it to assess whether 50 imputations were sufficient. If the number of imputations was greater than 100xFMI, then the MI provides an adequate level of reproducibility [35]. We conducted all analyses in STATA 14.0 (College Station, Texas) and 95% confidence intervals (CI) were calculated.

## Results

There were 1121 participants with valid measures of PWV and AI, of whom 60% were women. Characteristics of individuals who were lost to follow-up and those who participated in the 2015–17 follow-up study are described elsewhere [31].

No significant differences in PWV were found between men (7.6±1.1 m/s) and women (7.6±1.3 m/s), however women had significantly higher AI (34.4±9.6%) compared to men (23.0±9.8%). Despite the high prevalence of hypertension among the population, particularly in women (44%) compared to men (36%); less than 10% receive hypertension medication. Overall women tended to have higher values of current cardiometabolic risk factors compared to men (Table 1).

**Table 1:** General characteristics of the study population, by sex.

Characteristics	Women (N = 674)	Men (N = 447)
Age at follow-up (y)	45.0±4.3	44.8±4.2
*Atole* supplementation during the first 1000 days of life^a^		
Unexposed (%)	14.8	13.6
Partial exposure, (%)	18.0	16.8
Full exposure, (%)	22.1	22.4
Childhood household socioeconomic status		
Poorest (%)	34.5	32.2
Middle (%)	32.3	33.5
Wealthiest (%)	33.3	34.2
Maternal age (y)	26.8 ±7.1	27.0±7.3
Maternal height (cm)	148.4±5.1	148.5±4.9
Maternal schooling (y)	1.2±1.5	1.4±1.6
Participant’s schooling (y)	3.3±2.1	3.5±2.1
Current socioeconomic status, N (%)		
Poorest, (%)	32.9	33.3
Middle, (%)	34.4	30.4
Wealthiest (%)	32.6	36.2
Height (cm)	151.5±5.3	163.9±6.1
Body mass index–BMI- (kg/m^2^)	29.2±5.1	26.6±4.3
Obesity^b^ (%)	39.6	19.2
Body fat (%)	42.2±5.9	28.8±6.7
Fat free mass (Kg)	38.3±5.6	50.5±6.9
Waist-to-height ratio^c^	0.67±0.03	0.57±0.03
Tobacco use (%)	0.9	30.4
Alcohol use (%)	4.3	37.0
Total cholesterol (mg/dL)	190.4±39.0	179.5±38.7
High density lipoprotein cholesterol (mg/dL)	37.2±11.4	34.1±11.0
Low density lipoprotein cholesterol (mg/dL)	117.1±35.8	107.3±36.6
Triglycerides (mg/dL)	226.5±112.2	242.9±155.3
Systolic blood pressure (mmHg)	124.5±18.0	123.7±13.7
Diastolic blood pressure (mmHg)	74.5±10.6	73.1±9.1
Mean blood pressure^d^ (mmHg)	91.2±012.4	89.9±10.1
Hypertension^e^ (%)	44.0	36.0
Hypertension medication (Yes, %)	11.7	3.1
Type 2 diabetes^f^ (%)	21.0	13.0
Heart rate (bpm)	68.3±9.68	61.9±9.5
Augmentation index^g^ (%)	34.3±9.6	23.0±9.8
Augmentation index at 75 bpm (%)	31.2±9.6	16.8±10.7
Augmentation index, % (AI > 0)	34.3±9.6	23.3±9.5
Pulse wave velocity–PWV (m/s)	7.6±1.3	7.6±1.1

^a^Full exposure: children who received full supplementation with *Atole* during the first 1000 days. Partial exposure: Children who received partial supplementation with *Atole* during the first 1000 days. Unexposed: children who received *Fresco* and those who received *Atole* outside the first 1000 days.

^b^Obesity: BMI ≥30 kg/m^2^ [19].

^c^Waist-to-height ratio: Waist circumference/ height in meters.

^d^Mean blood Pressure = diastolic blood pressure + 1/3 (systolic blood pressure–diastolic blood pressure).

^e^Hypertension: systolic blood pressure ≥130 mmHg and/or diastolic blood pressure ≥80 mmHg and/or hypertension medication [22].

^f^ Type 2 diabetes: fasting plasma glucose ≥126 mg/dL, and/or 2-h post-challenge glucose ≥200 mg/dL, and/or diabetes medication [24].

Bpm: beats per minute.

Sample size for variables with missing information for women and men respectively: Childhood household SES = 612 and 412; maternal height = 537 and 353; maternal schooling = 656 and 425; body fat = 656 and 435; fat free mass = 657 and 436; tobacco use = 673 (only women); alcohol use = 673 (only women); blood lipids = 667 and 435; blood pressure = 673 (only women); hypertension medication = 673 and 446; diabetes, = 444 (only men).

Medians and selected percentiles of PWV and AI by sex and age and their corresponding references values are presented in Table 2. Overall, this population has PWV values that were 11–16% lower than the reference values. In the groups of 37y-45y and 45y-55y, women have higher AI (50^th^ percentile: 33 and 36.0%) compared with the median of age-specific reference values (50^th^ percentile: 22.2 and 26.8%) (Table 2). In men, the median AI were close to the reference values (<10% difference).

**Table 2:** Percentiles of pulse wave velocity and augmentation index by sex and age-group in the 2015–17 follow-up of the INCAP Nutrition Supplementation Trial Longitudinal Study.

Sex	Sample size	Women (n = 674)			Men (n = 447)			Reference values		
	n	10^th^	50th	90th	10th	50^th^	90th	10th	50th	90th
Pulse wave velocity^a^ (m/s)										
37-45y	630	6.1	7.2	8.8	6.3	7.3	8.6	6.6	8.6	11.0
46-55y	499	6.2	7.9	9.6	6.4	7.6	9.2	6.7	8.9	10.1
Augmentation index^b^ (%)										
37-45y	630	21	33	47	10	20	35	-	22.2	-
46-55y	499	25	36	47	13	26	37	-	26.8	-

^a^ Pulse wave velocity references derived from a Meta-analysis of 8167 participants. References correspond to 10th, 50^th^ y 90^th^ percentile of an average of sex and for 40y - 50y respectively [26].

^b^ Augmentation index references derived from 4,561 adults in a cohort without known cardiovascular disease (CVD) or diabetes, and with low risk of CVD from The Copenhagen City Heart Study (SphygmoCor device). References correspond for an average of sex and for 40y - 50y respectively using the following equations Men: 79.20 + 0.63 (age) − 0.002 (age^2^) − 0.28 (heart rate) − 0.39 (height). Women: AI = 56.28 + 0.90 (age) − 0.005 (age^2^) − 0.34 (heart rate) − 0.24 (height) [27].

AI and PWV were higher in those with obesity compared with participants without obesity (men: 7.02% 95% CI: 4.58, 9.46 and 0.50 m/s 95% CI: 0.24, 0.76; women: 2.32% 95% CI: 0.85, 3.80 and 0.60 m/s 95% CI: 0.41, 0.81, respectively; data in S1 Table). Height was inversely associated with AI but positively associated with PWV in both men and women. Current SES was positively associated with PWV in men. Women living in Guatemala City had lower AI (-2.11%; 95% CI -3.98, -0.24) compared with women living in other locations. In contrast, men who lived in Guatemala City had higher PWV compared with those living in other locations. PWV and AI were also positively associated with age and with several other cardio-metabolic risk factors.

In the analysis of the association between early life supplementation and arterial stiffness in adulthood, there was no evidence of heterogeneity of associations by gender; hence, we focus on the results of the pooled, sex-adjusted models (Table 3). Sex-stratified estimates are provided in S2 Table. Models 1, 2, and 3 did not differ meaningfully. Hence, we focus on findings from model 3, which was adjusted for the baseline and current sociodemographic factors.

**Table 3:** The association of early life nutrition^a^ during the first 1000 days of life with pulse wave velocity and augmentation index in adulthood, INCAP Nutrition Supplementation Trial Longitudinal Study.

	Full or partial exposure vs unexposed		Full exposure vs. unexposed		Partial exposure vs. unexposed	
Measure of arterial stiffness	β ^b^	95% CI	β	95% CI	β	95% CI
Pulse wave velocity, m/s						
Model 1^c^	-0.31	[-0.83,0.20]	-0.22	[-0.79,0.34]	-0.33	[-0.89,0.24]
Model 2^d^	-0.32	[-0.83,0.20]	-0.22	[-0.79,0.35]	-0.32	[-0.89,0.24]
Model 3^e^	-0.36*	[-0.79,0.08]	-0.39*	[-0.87,0.09]	-0.28	[-0.76,0.20]
Model 4^f^	-0.40*	[-0.83,0.02]	-0.45*	[-0.93,0.01]	-0.32	[-0.77,0.08]
Augmentation index, %						
Model 1	0.36	[-3.79, 4.51]	1.13	[-3.51,5.76]	-0.35	[-4.95,4.26]
Model 2	0.31	[-3.85, 4.47]	1.04	[-3.61,5.70]	-0.37	[-4.99,4.26]
Model 3	0.33	[-3.79, 4.45]	0.90	[-3.70,5.51]	-0.21	[-4.78,4.36]
Model 4	-0.21	[-4.23,3.80]	0.21	[-4.30,4.71]	-0.58	[-5.03,3.87]
Augmentation index at 75 bpm, %						
Model 1	0.08	[-4.37,4.52]	1.19	[-3.80,6.17]	-1.29	[-6.25,3.66]
Model 2	-0.11	[-4.59,4.37]	1.18	[-3.83,6.18]	-1.29	[-6.26,3.69]
Model 3	-0.14	[-4.48,4.19]	0.73	[-4.11,5.57]	-0.98	[-5.78,3.83]
Model 4	-0.69	[-4.93,3.54]	0.06	[-4.68,4.81]	-1.39	[-6.08,3.30]

*p ≤ 0.1. Sample size: 447 men, 674 women.

^a^Full exposure: children who received full supplementation with *Atole* during the first 1000 days. Partial exposure: children who received partial supplementation with *Atole* during the first 1000 days. Unexposed: children who received *Fresco* and *Atole* outside the first 1000 days.

^b^Estimates are β coefficients of the interaction term between exposure period and specifying exposure to *Atole* from conception to age 2 y controlling for: fixed effects of birth village and supplementation type (*Atole* vs. *Fresco*), age at intervention (whole exposure vs other) and birth year.

^c^Model 1: Interaction term (supplementation type and exposure period), birth year and heart rate.

^d^Model 2: Model 1 + maternal height, maternal schooling, maternal age at the birth of child and childhood household socioeconomic status.

^e^Model 3. Additional adjustment for current age, socioeconomic status, residency, use of tobacco, alcohol, hypertension medication, mean blood pressure and height.

^f^Model 4. Additional adjustment for waist-to-height-ratio, triglycerides, high-density lipoprotein, low-density lipoprotein, BMI, fat-free mass, body fat percentage and diabetes.

In model 3, we found lower PWV in individuals with full exposure to *Atole* during the first 1000 days (-0.39m/s, 95% CI -0.87, 0.09; p = 0.10) compared with those who were unexposed. We observed a smaller estimate in individuals with partial exposure to *Atole* compared with unexposed children (-0.28m/s, 95% CI -0.76, 0.20; p = 0.17). PWV was amplified when adjusting for cardiometabolic risk factors (-0.45m/s; 95%C-0.93, 0.01; p = 0.06) in the full exposure group. Neither partial nor full exposure to *Atole* during the first 1000 days was significantly associated with AI (-0.21%, 95% CI -4.78, 4.36 and 0.90%, 95%CI -3.70, 5.51, respectively). Standardizing AI to 75bpm did not change the estimates. Overall, in all AI models, an attenuation of the estimates was observed following adjustment for other cardiometabolic disease risk markers (Table 3 and S2 Table).

We also examined whether any exposure to *Atole* in the first 1000 days was associated with AI by combining the full and partial categories (Table 3 and S2 Table). In this specification, any *Atole* supplementation during the first 1000 days was marginally associated with reduced PWV (-0.36 95%CI -0.79, 0.08; p = 0.10). This categorization of exposure was not significantly associated with AI.

## Discussion

Our main finding is that exposure to *Atole* supplementation during the first 1000 days is marginally associated with lower PVW in adulthood, independently of attained height, mean blood pressure and current cardiometabolic risk factors. Exposure to *Atole* during the first 1000 days was not associated with AI.

To date, the influence of early-life nutrition on arterial stiffness in adulthood is relatively unexplored. In the United Kingdom, a cohort of infants small for gestational age who were randomly assigned to receive a nutrient-enriched formula from birth to 9 m, showed more catch-up in weight and increased blood pressure in childhood compared with those who received standard formula [14]. Two studies in preterm babies found that those who received enriched formulas during the first month of life had higher blood pressure at 13–16 y compared to those who were breastfed [36]. However, our results might not be comparable with these studies due to differences on the type of supplementation, periods of exposure, pre-natal outcomes, and socio-economic backgrounds.

In countries with accelerated income growth, along with poor-quality diets, better known as nutrition transition, there is evidence on the association between growth failure and risk of elevated blood pressure in adulthood [16]. All studies to date had only explored the influence of early nutrition on peripheral blood pressure, and to our knowledge, this is the first study exploring the influence of early life nutrition on the burden of central blood pressure in a low and middle-income country.

Our participants are from a low-income, rural population in Guatemala who suffered from severe stunting early in life. Children who received *Atole* from conception to age 2 years had improved linear growth compared with those unexposed, even though stunting prevalence was 52% at 7 years old [37].

Several observational studies have documented that people with short stature may be more susceptible to the effects of arterial-tree aging [38–40]. Body height might affect arterial wave reflections since it reduces the timing between forward- and backward-travelling pressure waves. A shorter stature reduces both the travel path (the distance) and the travel time, resulting in an earlier return of reflected pressure waves in the proximal aorta, leading to increased wave reflections and central blood pressure augmentation [41]. Women tend to have worse prognosis of arterial stiffness associated with shorter height [42], a reduced carotid diameter, and differences in the left ventricular performance compared to men [43]. In our study population, women have shorter height and higher AI as compared to men; however, no difference on PWV was found between men and women.

In our analysis combining partial and full exposure to *Atole*, the estimates showed lower PWV; however, in sensitivity analysis we demonstrated that such reduction is explained by the effect of the full exposure of *Atole* supplementation from conception to age 2 years. One potential mechanism is that improved nutrition in a critical window of development improves endothelial function, which is an important regulator of arterial stiffness, both functionally and structurally [44–46]. These results suggest that PWV might be more sensitive to early nutritional influences than AI.

Augmentation index in late middle age was not associated with early life improved nutrition. One explanation is that a high prevalence of atherogenic risk factors, reduces variance and limits the power to detect associations. Participants in our study have a poor cardiometabolic profile compared with several other populations of similar age [38, 47]. Particularly in women, the atherogenic profile (blood lipids, AI and hypertension prevalence) is similar to the levels observed in older populations [26, 48]. Recent analysis showed that *Atole* supplementation was positively associated with body fatness and obesity, all potential mediators of AI [31]. In the analysis of AI, the inclusion of potential mediators produced an attenuation of the estimates, contrary to what was observed using PWV, suggesting that AI might be more influenced by determinants of atherosclerosis; however, it is still controversial whether AI is a surrogate measure of arterial stiffness or an index of wave reflection [49–51]. This data might support the argument that AI and PWV, as measures of arterial stiffness, are not interchangeable [49, 52]; however, further research is needed to confirm these results and to determine whether different pathways are involved in the onset of increased AI and PWV.

Limitations of the study might include residual confounding. We could not examine the specific role of breastfeeding [12], since breastfeeding was a universal practice in rural Guatemala in past decades [53]. Birth weight, an indicator of children who have suffered growth retardation in utero, has been proposed as a potential confounder since previous studies suggest that atherosclerosis and cardiovascular outcomes in adulthood starts in utero [54, 55]. Studies have observed that low birth weight is inversely associated with atherosclerosis as well as cardiovascular morbidity and mortality in later life [56, 57]. Our study does not allow us to examine the role of birthweight as many of the children unexposed to *Atole* supplementation were born before the intervention started and birth records were not collected at that time. Another potential limitation is attrition; however, in previous analyses we have shown that attrition did not vary by exposure status [31]. In addition, available arterial stiffness reference values were constructed with European descent populations, limiting the comparability with this population. We also acknowledge that power considerations limit our ability to investigate further pathways.

Strengths of the current work is that the INCAP Nutrition Supplementation Trial Longitudinal Study offers a prospective follow-up of a large population-based cohort exposed to a nutritional intervention in early life. Another strength is that, to our knowledge, this is the first time that arterial stiffness is examined in Central America and there is no previous research into these measures in populations who experienced early growth failure.

In conclusion, improved nutrition in early life might reduce PWV, the gold standard to measure arterial stiffness, independently of cardiometabolic risk but has no association with AI. More studies are needed to confirm these results and to understand the role and mechanisms of early life nutrition on arterial stiffness.

## Supporting information

S1 Table Bivariate association between measures of pulse wave velocity, augmentation index and cardio-metabolic risk factors, age, residency and socioeconomic status by sex in the 2015–2017 follow-up of the INCAP Nutrition Supplementation Trial Longitudinal Study. *p<0.05, ** *p*<0.01, ***p<0.001. ^a^Estimates are β coefficients of the association between measures of Arterial Stiffness and co-variates by sex. ^b^Augmentation index: Augmentation pressure /pulse pressure. Augmentation index was standardized at 75bpm. ^c^Obesity: BMI ≥30 kg/m^2^ [19]. ^d^Association of arterial stiffness measures with alcohol and smoking status performed only in men. ^e^Waist-to-height ratio used as a dichotomous variable to define central obesity: ≥ 0.5 (Waist circumference/ height in meters) [20]. ^f^Type 2 diabetes: fasting plasma glucose ≥126 mg/dL, and/or 2-h post-challenge glucose ≥200 mg/dL, and/or diabetes medication use [24]. ^g^Mean blood pressure = diastolic blood pressure + 1/3 (systolic blood pressure–diastolic blood pressure). SES: Socioeconomic status, BMI: body mass index, HDL- high density lipoprotein cholesterol, LDL low density lipoprotein cholesterol, SBP: systolic blood pressure, DBP: diastolic blood pressure, MBP: mean blood pressure.
(DOCX)

S2 Table Sex-specific associations between early life nutrition during the first 1000 days of life and pulse wave velocity and augmentation index in adulthood based on different levels of *Atole* exposure in the 2015–2017 follow-up of the INCAP Nutrition Supplementation Trial Longitudinal Study. ^a^Full exposure: children who received full supplementation with *Atole* during the first 1000 days of life. Partial exposure: Children who received partial supplementation with *Atole* during the first 1000 days. Unexposed: children who received Fresco and those who receive *Atole* outside the first 1000 days. ^b^Estimates are *B* coefficients of the interaction term between exposure period and specifying exposure to *Atole* from conception to age 2 y controlling for: fixed effects of birth village and supplementation type (*Atole* vs. *fresco*), age at intervention (whole exposure vs other) and birth year. All models are adjusted by heart rate. ^c^Model 1. Interaction term (supplementation type and exposure period) and birth year. ^d^ Model 2. Model 1 + maternal Height, maternal schooling, maternal age at the birth of child and childhood household socioeconomic status. ^e^ Model 3. Additional adjustment for current age, socioeconomic status, residency, use of tobacco, alcohol, hypertension medication, mean blood pressure and height. ^f^ Model 4. Additional adjustment for waist-to-height ratio, triglycerides, high-density lipoprotein, low-density lipoprotein, body mass index, fat-free mass, body fat percentage and type 2 diabetes diagnosis. ^g^Augmentation index: Augmentation pressure /pulse pressure. bpm: beat per minute.
(DOCX)

## References

[1] Payne RA, Wilkinson IB, Webb DJ. Arterial stiffness and hypertension: Emerging concepts. Hypertension. 2010 pp. 9–14. 10.1161/HYPERTENSIONAHA.107.090464 19948990

[2] McEniery CM, Cockcroft JR, Roman MJ, Franklin SS, Wilkinson IB. Central blood pressure: Current evidence and clinical importance. Eur Heart J. 2014;35 10.1093/eurheartj/eht565 24459197 PMC4155427

[3] Van Popele NM, Grobbee DE, Bots ML, Asmar R, Topouchian J, Reneman RS, et al Association between arterial stiffness and atherosclerosis: The Rotterdam study. Stroke. 2001;32: 454–460. 10.1161/01.str.32.2.454 11157182

[4] Vlachopoulos C, Aznaouridis K, O’Rourke MF, Safar ME, Baou K, Stefanadis C. Prediction of cardiovascular events and all-cause mortality with central haemodynamics: a systematic review and meta-analysis. Eur Heart J. 2010;31: 1865–1871. 10.1093/eurheartj/ehq024 20197424

[5] Zhong Q, Hu M-J, Cui Y-J, Liang L, Zhou M-M, Yang Y-W, et al Carotid–Femoral Pulse Wave Velocity in the Prediction of Cardiovascular Events and Mortality: An Updated Systematic Review and Meta-Analysis. Angiology. 2018;69: 617–629. 10.1177/0003319717742544 29172654

[6] Brunner EJ, Shipley MJ, Ahmadi-Abhari S, Tabak AG, Mceniery CM, Wilkinson IB, et al Adiposity, Obesity, and Arterial Aging: Longitudinal Study of Aortic Stiffness in the Whitehall II Cohort. Hypertension. 2015;66: 294–300. 10.1161/HYPERTENSIONAHA.115.05494 26056335 PMC4490910

[7] Ecobici M, Stoicescu C. Arterial Stiffness and Hypertension—Which Comes First? Maedica (Buchar). 2017. PMC5706758 29218066

[8] Canepa M, AlGhatrif M, Pestelli G, Kankaria R, Makrogiannis S, Strait JB, et al Impact of central obesity on the estimation of carotid-femoral pulse wave velocity. Am J Hypertens. 2014;27: 1209–1217. 10.1093/ajh/hpu038 24637912 PMC4141203

[9] Johansen NB, Vistisen D, Brunner EJ, Tabák AG, Shipley MJ, Wilkinson IB, et al Determinants of aortic stiffness: 16-year follow-up of the Whitehall II study. PLoS One. 2012;7: 1–8. 10.1371/journal.pone.0037165 22629363 PMC3358295

[10] Saeedi P, Shavandi A, Skidmore P, Saeedi P, Shavandi A, Skidmore PML. What Do We Know about Diet and Markers of Cardiovascular Health in Children: A Review. Int J Environ Res Public Health. 2019;16: 548 10.3390/ijerph16040548 30769798 PMC6406429

[11] Järvisalo MJ, Hutri-Kähönen N, Juonala M, Mikkilä V, Räsänen L, Lehtimäki T, et al Breast feeding in infancy and arterial endothelial function later in life. The Cardiovascular Risk in Young Finns Study. Eur J Clin Nutr. 2009;63: 640–645. 10.1038/ejcn.2008.17 18285807

[12] Martin RM, Ebrahim S, Griffin M, Smith GD, Nicolaides AN, Georgiou N, et al Breastfeeding and Atherosclerosis. Arterioscler Thromb Vasc Biol. 2005;25: 1482–1488. 10.1161/01.ATV.0000170129.20609.49 15890972

[13] Evelein Mv A, Geerts C. C, Visseren Lj F, Bots L. M, van der Ent K. C, Grobbee E. D, et al The association between breastfeeding and the cardiovascular system in early childhood. Am J Clin Nutr. 2011;93: 712–718. 10.3945/ajcn.110.002980 21310835

[14] Singhal A, Cole TJ, Fewtrell M, Kennedy K, Stephenson T, Elias-Jones A, et al Promotion of Faster Weight Gain in Infants Born Small for Gestational Age Is There an Adverse Effect on Later Blood Pressure? Circulation. 2007;115: 213–220. 10.1161/CIRCULATIONAHA.106.617811 17179023

[15] Martin R, McCarthy A, Smith G, Davies D, Ben-Shlomo Y. Infant nutrition and blood pressure in early adulthood: the Barry Caerphilly Growth study. Am J Clin Nutr. 2003;77: 1489–1497 9p. 10.1093/ajcn/77.6.1489 12791629

[16] Stein AD, Thompson AM, Waters A. Childhood growth and chronic disease: evidence from countries undergoing the nutrition transition. Matern Child Nutr. 2005;1: 177–184. 10.1111/j.1740-8709.2005.00021.x 16881898 PMC6860951

[17] Martorell R, Jean-Pierre H, Rivera JA. History and design of the INCAP longitudinal study (1969–77) and its follow-up (1988–89). J Nutr. 1995; 1027S–1041S. 10.1093/jn/125.suppl_4.1027S 7536830

[18] Lohman TG, Roche A, Martorell R. Anthropometric standardization reference manual. Champaign IL: Human Kinetics Books; 1988 Available: http://www.worldcat.org/title/anthropometric-standardization-reference-manual/oclc/15592588

[19] National Heart Lung and Blood Institute Obesity Education Initiative. The Practical Guide. Identification, Evaluation, and Treatment of Overweight and Obesity in Adults. National Institutes of Health 2000 10.1016/j.physleta.2015.01.006

[20] Ashwell M, Gunn P, Gibson S. Waist-to-height ratio is a better screening tool than waist circumference and BMI for adult cardiometabolic risk factors: Systematic review and meta-analysis. Obesity Reviews. 2012 pp. 275–286. 10.1111/j.1467-789X.2011.00952.x 22106927

[21] Van Marken Lichtenbelt WD, Westerterp KR, Wouters L. Deuterium dilution as a method for determining total body water: effect of test protocol and sampling time. Br J Nutr. 2007/03/01. 1994;72: 491–497. 10.1079/bjn19940053 7986782

[22] Reboussin DM, Allen NB, Griswold ME, Guallar E, Hong Y, Lackland DT, et al Systematic Review for the 2017 ACC/AHA/AAPA/ABC/ACPM/AGS/APhA/ASH/ASPC/NMA/PCNA Guideline for the Prevention, Detection, Evaluation, and Management of High Blood Pressure in Adults. J Am Coll Cardiol. 2018;71: 2176–2198. 10.1016/j.jacc.2017.11.004 29146534 PMC8654280

[23] Sainas G, Milia R, Palazzolo G, Ibba G, Marongiu E, Roberto S, et al Mean Blood Pressure Assessment during Post-Exercise: Result from Two Different Methods of Calculation. J Sports Sci Med. 2016;15: 424–433. 27803621 PMC4974855

[24] American Diabetes Association. Classification and diagnosis of diabetes. Diabetes Care. 2017;40: S11–S24. 10.2337/dc17-S005 27979889

[25] Martorell R. Improved nutrition in the first 1000 days and adult human capital and health. Am J Hum Biol. 2017;29 10.1002/ajhb.22952 28117514 PMC5761352

[26] Khoshdel AR, Thakkinstian A, Carney SL, Attia J. Estimation of an age-specific reference interval for pulse wave velocity: A meta-analysis. J Hypertens. 2006;24: 1231–1237. 10.1097/01.hjh.0000234098.85497.31 16794467

[27] Janner JH, Godtfredsen NS, Ladelund S, Vestbo J, Prescott E. Aortic augmentation index: Reference values in a large unselected population by means of the sphygmocor device. Am J Hypertens. 2010;23: 180–185. 10.1038/ajh.2009.234 19959999

[28] John A. Maluccio, Alexis Murphy and KMY. Research Note: A socioeconomic index for the INCAP Longitudinal Study 1969–77. Food Nutr Bull. 2005;26. 10.1177/15648265050262S112 16060218

[29] Stein AD, Wang M, DiGirolamo A, Grajeda R, Ramakrishnan U, Ramirez-Zea M, et al Nutritional supplementation in early childhood, schooling, and intellectual functioning in adulthood: A prospective study in guatemala. Arch Pediatr Adolesc Med. 2008 10.1001/archpedi.162.7.612 18606931 PMC3733080

[30] Hoddinott J, Maluccio JA, Behrman JR, Flores R, Martorell R. Effect of a nutrition intervention during early childhood on economic productivity in Guatemalan adults. Lancet. 2008;371: 411–416. 10.1016/S0140-6736(08)60205-6 18242415

[31] Ford ND, Martorell R, Stein AD, Behrman JR, Dphil H, Maluccio JA, et al Exposure to improved nutrition from conception to age 2 years and adult cardiometabolic disease risk: a modelling study. Lancet Glob Heal. 2018;6: e875–e884. 10.1016/S2214-109X(18)30231-6 30012268 PMC6138451

[32] Lantelme P, Mestre C, Lievre M, Gressard A, Milon H. Heart rate: An important confounder of pulse wave velocity assessment. Hypertension. 2002;39: 1083–1087. 10.1161/01.hyp.0000019132.41066.95 12052846

[33] Hughes AD, Park C, Davies J, Francis D, McG Thom SA, Mayet J, et al Limitations of Augmentation Index in the Assessment of Wave Reflection in Normotensive Healthy Individuals. PLoS One. 2013;8 10.1371/journal.pone.0059371 23544061 PMC3609862

[34] Hayati Rezvan P, Lee KJ, Simpson JA. The rise of multiple imputation: a review of the reporting and implementation of the method in medical research. BMC Med Res Methodol. 2015;15: 30 10.1186/s12874-015-0022-1 25880850 PMC4396150

[35] Little RJ, Wang J, Sun X, Tian H, Suh E-Y, Lee M, et al The treatment of missing data in a large cardiovascular clinical outcomes study. Clin Trials. 2016;13: 344–351. 10.1177/1740774515626411 26908543

[36] Singhal A, Cole TJ, Lucas A. Early nutrition in preterm infants and later blood pressure: two cohorts after randomised trials. Lancet (London, England). 2001;357: 413–9. 10.1016/S0140-6736(00)04004-6 11273059

[37] Corvalán C, Gregory CO, Ramirez-Zea M, Martorell R, Stein AD. Size at birth, infant, early and later childhood growth and adult body composition: a prospective study in a stunted population. Int J Epidemiol. 2007;36: 550–557. 10.1093/ije/dym010 17376801

[38] Kannam JP, Levy D, Larson M, Wilson PWF. Short stature and risk for mortality and cardiovascular disease events: The Framingham Heart Study. Circulation. 1994;90: 2241–2247. 10.1161/01.cir.90.5.2241 7955180

[39] Korhonen PE, Kautiainen H, Eriksson JG. The shorter the person, the higher the blood pressure: A birth cohort study. J Hypertens. 2017;35: 1170–1177. 10.1097/HJH.0000000000001300 28441691

[40] Smulyan H, Marchais SJ, Pannier B, Guerin AP, Safar ME, London GM. Influence of Body Height on Pulsatile Arterial Hemodynamic Data. J Am Coll Cardiol. 1998;31: 1103–1109. 10.1016/s0735-1097(98)00056-4 9562014

[41] Langenberg C, Hardy R, Breeze E, Kuh D, Wadsworth MEJ. Influence of short stature on the change in pulse pressure, systolic and diastolic blood pressure from age 36 to 53 years: An analysis using multilevel models. Int J Epidemiol. 2005;34: 905–913. 10.1093/ije/dyi071 15833796

[42] Wamala SP, Mittleman MA, Horsten M, Schenck-Gustafsson K, Orth-Gomér K. Short stature and prognosis of coronary heart disease in women. J Intern Med. 1999;245: 557–563. 10.1046/j.1365-2796.1999.00454.x 10395184

[43] Gatzka CD, Kingwell BA, Cameron JD, Berry KL, Liang YL, Dewar EM, et al Gender differences in the timing of arterial wave reflection beyond differences in body height. J Hypertens. 2001;19: 2197–2203. 10.1097/00004872-200112000-00013 11725164

[44] Oliver JJ, Webb DJ. Noninvasive assessment of arterial stiffness and risk of atherosclerotic events. Arterioscler Thromb Vasc Biol. 2003;23: 554–566. 10.1161/01.ATV.0000060460.52916.D6 12615661

[45] Waterland RA, Garza C. Potential mechanisms of metabolic imprinting that lead to chronic disease. Am J Clin Nutr. 1999;69: 179–97. 10.1093/ajcn/69.2.179 9989679

[46] LaRocca TJ, Martens CR, Seals DR. Nutrition and other lifestyle influences on arterial aging. Ageing Res Rev. 2017;39: 106–119. 10.1016/j.arr.2016.09.002 27693830 PMC5373983

[47] Lajous M, Ortiz-Panozo E, Monge A, Santoyo-Vistrain R, García-Anaya A, Yunes-Díaz E, et al Cohort Profile: The Mexican Teachers’ Cohort (MTC). Int J Epidemiol. 2015;46: e10(1–10) 10.1093/ije/dyv123 26337903

[48] Duprez DA, Jacobs DR, Lutsey PL, Herrington D, Prime D, Ouyang P, et al Race/ethnic and sex differences in large and small artery elasticity—results of the multi-ethnic study of atherosclerosis (MESA). Ethn Dis. 2009;19: 243–50. 19769004 PMC2924814

[49] Sakurai M, Yamakado T, Kurachi H, Kato T, Kuroda K, Ishisu R, et al The relationship between aortic augmentation index and pulse wave velocity: an invasive study. J Hypertens. 2007;25: 391–397. 10.1097/HJH.0b013e3280115b7c 17211246

[50] Lemogoum D, Flores G, Van den Abeele W, Ciarka A, Leeman M, Degaute JP, et al Validity of pulse pressure and augmentation index as surrogate measures of arterial stiffness during beta-adrenergic stimulation. J Hypertens. 2004;22: 511–7. 10.1097/00004872-200403000-00013 15076156

[51] Lacy PS, O’Brien DG, Stanley AG, Dewar MM, Swales PPR, Williams B. Increased pulse wave velocity is not associated with elevated augmentation index in patients with diabetes. J Hypertens. 2004;22: 1937–44. 10.1097/00004872-200410000-00016 15361765

[52] Jerrard-Dunne P, Mahmud A, Feely J. Ambulatory arterial stiffness index, pulse wave velocity and augmentation index—Interchangeable or mutually exclusive measures? J Hypertens. 2008;26: 529–534. 10.1097/HJH.0b013e3282f35265 18300865

[53] Merchant K, Martorell R, Hass J. Maternal and fetal responses to the stresses of lactation with pregnancy and of short recuperative intervals. Am J Clin Nutr. 1990;52: 280–8. 10.1093/ajcn/52.2.280 2375294

[54] Martyn CN, Gale CR, Jespersen S, Sherriff SB. Impaired fetal growth and atherosclerosis of carotid and peripheral arteries. Lancet. 1998;352: 173–178. 10.1016/S0140-6736(97)10404-4 9683205

[55] Barker DJ. The fetal and infant origins of disease. Eur J Clin Invest. 1995;25: 457–463. 10.1111/j.1365-2362.1995.tb01730.x 7556362

[56] Leon DA, Lithell HO, Vâgerö D, Koupilová I, Mohsen R, Berglund L, et al Reduced fetal growth rate and increased risk of death from ischaemic heart disease: cohort study of 15 000 Swedish men and women born 1915–29. BMJ. 1998;317: 241–5. 10.1136/bmj.317.7153.241 9677213 PMC28614

[57] Barker DJ., Osmond C, Winter P., Margetts B, Simmonds S. Weight in infancy and death from ischaemic heart disease. Lancet. 1989;334: 577–580. 10.1016/S0140-6736(89)90710-1 2570282

