# Supplementary material for: Improved nutrition in early life and pulse wave velocity and augmentation index in mid-adulthood: Follow-up of the INCAP Nutrition Supplementation Trial Longitudinal Study
Source: PLoS One. 2020 Oct 27;15(10):e0239921. doi: 10.1371/journal.pone.0239921 (PMC7591084; doi:10.1371/journal.pone.0239921)
Supplement: S1 Table — *p<0.05, ** p<0.01, ***p<0.001. aEstimates are β coefficients of the association between measures of Arterial Stiffness and co-variates by sex. bAugmentation index: Augmentation pressure /pulse pressure. Augmentation index was standardized at 75bpm. cObesity: BMI ≥30 kg/m2 [19]. dAssociation of arterial stiffness measures with alcohol and smoking status performed only in men. eWaist-to-height ratio used as a dichotomous variable to define central obesity: ≥ 0.5 (Waist circumference/ height in meters) [20]. fType 2 diabetes: fasting plasma glucose ≥126 mg/dL, and/or 2-h post-challenge glucose ≥200 mg/dL, and/or diabetes medication use [24]. gMean blood pressure = diastolic blood pressure + 1/3 (systolic blood pressure–diastolic blood pressure). SES: Socioeconomic status, BMI: body mass index, HDL- high density lipoprotein cholesterol, LDL low density lipoprotein cholesterol, SBP: systolic blood pressure, DBP: diastolic blood pressure, MBP: mean blood pressure. (DOCX) [file pone.0239921.s001.docx]

**S1 Table. Bivariate association between measures of pulse wave velocity , augmentation index and cardio-metabolic risk factors, age, residency and socioeconomic status by sex in the 2015-2017 follow-up of the INCAP Nutrition Supplementation Trial Longitudinal Study**

|  | Women (n=674) | | | | Men (n=447) | | | |
| --- | --- | --- | --- | --- | --- | --- | --- | --- |
|  | Pulse Wave Velocity , m/s | | Augmentation Index^b,^ % | | Pulse Wave Velocity, m/s | | Augmentation Index, % | |
| Co-Variates | β^a^ | [95% CI] | β | [95% CI] | β | [95% CI] | β | [95% CI] |
| Age, y | 0.08*** | [0.06,0.11] | 0.30*** | [0.14,0.47] | 0.05*** | [0.02,0.07] | 0.56*** | [0.32,0.79] |
| Current socioeconomic status, z-score | -0.01 | [-0.11,0.08] | -0.15 | [-0.89,0.58] | 0.18*** | [0.08,0.28] | 0.89 | [-0.85,1.86] |
| Residency (Guatemala City/other) | -0.01 | [-0.26,0.25] | -2.11* | [-3.98,-0.24] | 0.41** | [0.15,0.68] | 0.56 | [-1.98,3.10] |
| Body Mass Index, Kg/m^2^ | 0.06*** | [0.04,0.08] | 0.21** | [0.07,0.35] | 0.07*** | [0.04,0.09] | 0.88*** | [0.66,1.09] |
| Obesity^c^ (yes/no) | 0.60*** | [0.41,0.81] | 2.32*** | [0.85,3.80] | 0.50*** | [0.24,0.76] | 7.02*** | [4.58,9.46] |
| Smoking^d^ (men) | N/A |  | N/A |  | 0.34** | [0.11,0.56] | 1.69 | [-0.45,3.85] |
| Alcohol^d^ (men) | N/A |  | N/A |  | 0.22* | [0.01,0.44] | 2.56* | [0.52,4.61] |
| Fat-free mass, Kg | 0.04*** | [0.03,0.06] | -0.09 | [-0.22,0.04] | 0.04*** | [0.02,0.05] | 0.21** | [0.06,0.35] |
| Body Fat % | 0.05*** | [0.03,0.06] | 0.24*** | [0.12,0.36] | 0.03*** | [0.02,0.05] | 0.38*** | [0.24,0.52] |
| Height, mt | 0.03** | [0.01,0.05] | -0.28*** | [-0.42,-0.14] | 0.03** | [0.01,0.04] | -0.25** | [-0.42,-0.09] |
| Waist-to-height ratio^e^ (>0.5) | 1.04 | [-0.25,2.34] | 8.52 | [-0.94,17.98] | 0.55*** | [0.22,0.87] | 6.27*** | [3.20,9.34] |
| Type 2 Diabetes^f^(yes/No) | 0.86*** | [0.62,1.10] | 0.92 | [-0.86,2.71] | 0.75*** | [0.45,1.10] | 2.11 | [-0.83,5.06] |
| Peripheral Systolic Blood Pressure (mmHg) | 0.04*** | [0.04,0.05] | 0.06** | [0.02,0.10] | 0.05*** | [0.04,0.05] | 0.19*** | [0.12,0.26] |
| Peripheral Diastolic Blood Pressure (mmHg) | 0.07*** | [0.06,0.08] | 0.06 | [-0.01,0.12] | 0.07*** | [0.06,0.08] | 0.29*** | [0.19,0.39] |
| Mean Blood pressure^h^ (mmHg) | 0.06*** | [0.05,0.07] | 0.07* | [0.01,0.12] | 0.06*** | [0.05,0.07] | 0.27*** | [0.18,0.37] |
| Total Cholesterol (mg/dL) | 0.01*** | [0.01,0.02] | -0.01 | [-0.02,0.16] | 0.01* | [0.01,0.02] | 0.04** | [0.01,0.06] |
| High density lipoprotein Cholesterol, (mg/dL) | -0.01 | [-0.01,0.01] | -0.05 | [-0.11,0.01] | -0.01 | [-0.01,0.01] | -0.12** | [-0.21,-0.03] |
| LDL Cholesterol(mg/dL) | 0.01** | [0.01,0.01] | -0.01 | [-0.03,0.01] | 0.01 | [-0.01,0.01] | 0.01 | [-0.02,0.03] |
| Triglycerides(mg/dL) | 0.01*** | [0.01,0.02] | 0.01* | [0.00,0.01] | 0.01** | [0.01,0.02] | 0.01*** | [0.00,0.02] |
| Central Systolic Blood Pressure(mmHg) | 0.05*** | [0.05,0.06] | 0.18*** | [0.14,0.23] | 0.40*** | [0.31,0.49] | 0.01** | [0.01,0.02] |
| Central Diastolic Blood Pressure (mmHg) | 0.08*** | [0.07,0.09] | 0.14*** | [0.07,0.21] | 0.09*** | [0.08,0.10] | 0.31*** | [0.18,0.43] |

**p*<0.05, ** *p*<0.01, ****p*<0.001.

^a^Estimates are β coefficients of the association between measures of arterial stiffness and co-variates by sex.

^b^Augmentation Index: Augmentation pressure /pulse pressure. Augmentation index was standardized at 75bpm

^c^Obesity: BMI ≥30 kg/m^2^(19)

^d^Association of arterial atiffness measures with alcohol and smoking status performed only in men.

^e^Waist to height ratio used as a dichotomous variable to define central obesity: ≥ 0.5 (Waist circumference/ height in meters)(20).

^f^ Type 2 Diabetes: fasting plasma glucose ≥126 mg/dL, and/or 2-h post-challenge glucose ≥200 mg/dL, and/or diabetes medication use (24)

^g^Mean blood pressure = Diastolic blood pressure + 1/3 (Systolic blood pressure – diastolic blood pressure)

SES: Socioeconomic status, BMI: body mass index, HDL: high density lipoprotein cholesterol, LDL: low density lipoprotein cholesterol, SBP: systolic blood pressure, DBP: diastolic blood pressure, MBP: mean blood pressure
