# Supplementary material for: Improved nutrition in early life and pulse wave velocity and augmentation index in mid-adulthood: Follow-up of the INCAP Nutrition Supplementation Trial Longitudinal Study
Source: PLoS One. 2020 Oct 27;15(10):e0239921. doi: 10.1371/journal.pone.0239921 (PMC7591084; doi:10.1371/journal.pone.0239921)
Supplement: S2 Table — aFull exposure: children who received full supplementation with Atole during the first 1000 days of life. Partial exposure: Children who received partial supplementation with Atole during the first 1000 days. Unexposed: children who received Fresco and those who receive Atole outside the first 1000 days. bEstimates are B coefficients of the interaction term between exposure period and specifying exposure to Atole from conception to age 2 y controlling for: fixed effects of birth village and supplementation type (Atole vs. fresco), age at intervention (whole exposure vs other) and birth year. All models are adjusted by heart rate. cModel 1. Interaction term (supplementation type and exposure period) and birth year. d Model 2. Model 1 + maternal Height, maternal schooling, maternal age at the birth of child and childhood household socioeconomic status. e Model 3. Additional adjustment for current age, socioeconomic status, residency, use of tobacco, alcohol, hypertension medication, mean blood pressure and height. f Model 4. Additional adjustment for waist-to-height ratio, triglycerides, high-density lipoprotein, low-density lipoprotein, body mass index, fat-free mass, body fat percentage and type 2 diabetes diagnosis. gAugmentation index: Augmentation pressure /pulse pressure. bpm: beat per minute. (DOCX) [file pone.0239921.s002.docx]

**S2 Table. Sex-specific associations between early life nutrition during the first 1000 days of life and pulse wave velocity and augmentation index in adulthood based on different levels of *Atole* exposure in the 2015-2017 follow-up of the INCAP Nutrition Supplementation Trial Longitudinal Study**

|  | Women (n=674) | | | | | | | Men (n= 447) | | | | | | | |
| --- | --- | --- | --- | --- | --- | --- | --- | --- | --- | --- | --- | --- | --- | --- | --- |
|  | Partial or full / vs unexposed | | Full exposure vs. unexposed | | Partial exposure vs. unexposed | | Partial or full / vs unexposed | | | Full exposure vs. unexposed | | Partial exposure vs. unexposed | | |  |
| Measure of arterial stiffness | β^b^ | 95% CI | β | 95% CI | β | 95% CI | Β | | 95% CI | β | 95% CI | β | 95% CI |  |  |
| Pulse Wave Velocity, m/s |  |  |  |  |  |  |  | |  |  |  |  |  |  |  |
| Model 1^c^ | -0.23 | [-0.87,0.41] | -0.16 | [-0.86,0.53] | -0.14 | [-0.84,0.57] | 0.35 | | [-0.52, 1.22] | 0.56 | [-0.36,1.48] | 0.02 | [-0.97,1.01] |  |  |
| Model 2^d^ | -0.14 | -[0.79,0.50] | -0.15 | [-0.85,0.56] | -0.12 | [-0.83,0.59] | 0.35 | | [-0.56, 1.27] | 0.56 | [-0.40,1.51] | 0.01 | [-1.04,1.06] |  |  |
| Model 3^e^ | -0.24 | [-0.82, 0.33] | -0.29 | [-0.93,0.33] | -0.17 | [-0.81,0.47] | 0.19 | | [-0.58, 0.97] | 0.37 | [-0.44,1.18] | -0.12 | [-1.03,0.79] |  |  |
| Model 4^f^ | -0.31 | [-0.89,0.27,] | -0.35 | [-0.98,0.29] | -0.26 | [-0.90,0.38] | 0.11 | | [-0.72, 0.94] | 0.29 | [-0.58,1.18] | -0.25 | [-1.2,0.70] |  |  |
| Augmentation Index^g^, % |  |  |  |  |  |  |  | |  |  |  |  |  |  |  |
| Model 1 | 0.71 | [-4.15,5.57] | 0.06 | (-5.25,5.38) | 1.20 | (-4.17,6.58) | -0.94 | | [-8.02,6.13] | -0.78 | [-8.32,6.76] | -0.96 | [-9.07,7.15] |  |  |
| Model 2 | 0.80 | [-4.11,5.71] | 0.15 | [-5.23,5.53] | 1.28 | [-4.14,6.72] | -0.93 | | [-8.87,6.52] | -0.78 | [-8.65,7.09] | -0.85 | [-9.43,7.72] |  |  |
| Model 3 | 1.07 | [-3.85,5.99] | 0.39 | [-4.98,5.77] | 1.63 | [-3.80,7.07] | -0.06 | | [-7.92,7.81] | 0.21 | [-8.12,8.55] | -0.09 | [-9.27,9.08] |  |  |
| Model 4 | 0.51 | [-4.38,5.40] | -0.25 | [-5.60,5.10] | 1.20 | [-4.23,6.60] | -5.12 | | [-12.99,2.75] | -5.37 | [-13.76,3.03] | -4.64 | [-13.82,4.53] |  |  |
| Augmentation Index at 75 bpm, % |  |  |  |  |  |  |  | |  |  |  |  |  |  |  |
| Model 1 | 0.81 | [-4.29,5.91] | 0.59 | [-4.98,6.16] | 0.83 | [-4.80,6.46] | -0.25 | | [-8.28,7.79] | 0.29 | [-8.25,8.84] | -0.85 | [-10.05,8.35] |  |  |
| Model 2 | 0.80 | [-4.34,5.95] | 0.59 | [-5.05,6.20] | 0.83 | [-4.87,6.53] | -1.11 | | [-9.47,7.25] | -0.49 | [-9.31,8.33] | -1.91 | [-11.52,7.70] |  |  |
| Model 3 | 0.76 | [-4.30,5.82] | 0.46 | [-5.09,6.00] | 0.93 | [-4.67,6.52] | 0.06 | | [-8.47,8.60] | 1.04 | [-7.97,10.05] | -1.38 | [-11.30,8.54] |  |  |
| Model 4 | 0.28 | [-4.78,5.34] | -0.05 | [-5.59,5.49] | 0.48 | [-5.11,6.08] | -5.32 | | [-13.8,3.19] | -5.08 | [-14.14,3.99] | -6.17 | [-16.10,3.76] |  |  |

^a^Full exposure: children who received full supplementation with *Atole* during the first 1000 days of life. Partial exposure: children who received partial supplementation with *Atole* during the first 1000 days of life. Unexposed: children who received *Fresco* and those who receive *Atole* outside the 1000 day window.

^b^Estimates are *B* coefficients of the interaction term between exposure period and specifying exposure to *Atole* from conception to age 2 y controlling for: fixed effects of birth village and supplementation type (*Atole* vs. *fresco*), age at intervention (whole exposure vs other) and birth year. All models are adjusted by heart rate.

^c^Model 1. Interaction term (supplementation type and exposure period) and birth year.

^d^ Model 2. Model 1 + maternal height, maternal schooling, maternal age at the birth of child and childhood household socioeconomic status.

^e^ Model 3. Additional adjustment for current age, socioeconomic status, residency, use of tobacco, alcohol, hypertension medication, mean blood pressure and height.

^f^ Model 4. Additional adjustment for waist to height ratio, triglycerides, high-density lipoprotein, low-density lipoprotein, body mass index, fat-free mass, body fat percentage and type 2 diabetes diagnosis

^g^Augmentation index : Augmentation pressure /pulse pressure.

bpm: beat per minute
